# The Role of MWCNTs in Enhancing the Foam Stability and Rheological Behavior of Cement Pastes That Contain Air-Entraining and Superplasticizer Admixtures

**DOI:** 10.3390/nano13243095

**Published:** 2023-12-07

**Authors:** Ina Pundienė, Jolanta Pranckevičienė

**Affiliations:** Laboratory of Concrete Technology, Institute of Building Materials, Vilnius Gediminas Technical University, Linkmenų Str. 28, 08217 Vilnius, Lithuania; jolanta.pranckeviciene@vilniustech.lt

**Keywords:** multi-walled carbon nanotubes, air-entraining admixtures, superplasticizer, rheology, foaming index

## Abstract

This research delves into the intricate dynamics between multi-walled carbon nanotubes (MWCNTs), air-entraining admixtures (AEAs), and a range of superplasticizers (SPs) in cementitious systems, shedding light on key aspects of construction material innovation. The focus is on how MWCNTs, AEAs, and specific SPs—namely, lignosulfonate (LS), polycarboxylate (PCE), and polyacrylate (PA)—influence the stability of foams and the viscosity and setting times of cement pastes. To assess the impacts of these components, we employed foam stability assessments, viscosity measurement techniques, electrical conductivity analysis, and evaluations of dispersion and setting times. Results indicate that MWCNTs enhance foam stability and viscosity, with the degree of improvement contingent on the type and concentration of SPs and the presence of AEAs. Notably, SPs, particularly PCE and PA, markedly influence the properties of cement paste, including increasing dispersion values and modulating setting times, especially when combined with MWCNTs and AEAs. The study concludes that strategically combining MWCNTs with specific SPs and AEAs alters the physical properties of cementitious materials significantly, underscoring the potential for customizing material design in the construction sector.

## 1. Introduction

Using foaming-agent air-entrained admixture (AEA) can provide more opportunities for developing non-load-bearing structural materials. In this type of material, AEA is typically utilized as a foaming agent. AEA lowers the surface tension of the water. An AEA molecule’s hydrophobic tail, typically exhibited as a hydrocarbon chain, attracts air bubbles, whereas the hydrophilic head of an AEA molecule tends to be in water [1,2]. Due to the AEA’s interaction with the cement and other solid components in the mixture, an inorganic shell is created around the cement-based materials’ voids. Structural materials, being lightweight cement-based materials due to air bubbles in the microstructure, are characterized by the following properties: low density, thermal insulation, anti-vibration and sound insulation, energy absorption, and fire resistance [3]. The structural materials have evolved with the rise of energy-efficient and environmentally friendly buildings, moving beyond wall insulation and subgrade backfilling to light embankment, bridge backfilling, airport blocking systems, enclosure structures, and other application issues [4].

During cement-paste mixing, AEA induces the formation of a system of tiny, nearly spherical air bubbles. The bubbles are larger than the capillary pores because capillary pores are typically less than 0.01 mm in diameter, with a high proportion being less than 0.003 mm [5]. In cement paste, air bubbles are naturally unstable. It is possible to produce foam concrete with various porosity parameters by varying the species of admixture and the intensity and duration of mixing. An increase in AEA concentration will improve foaming until the critical micelle concentration, at which a further increase did not produce more foam [1]. One crucial aspect of foam concrete’s air-gap structure is how it influences the material’s strength, density, and durability [6,7]. The advantages of introducing air to concrete include improved cohesiveness (leading to less bleed and segregation) and enhanced resistance to freeze–thaw degradation. Surface tension and AEA film elasticity are key factors in the stability of the foam. Research [8] indicates that the foam stability is increased in cement paste. There are four crucial prerequisites for the entrained air stability in concrete: the presence of air, less surface tension at the air/water interface, enough shell elasticity and strength at the air/water interface to prevent significant thinning during disturbance, and enough matrix viscosity to prevent bubble buoyancy [9]. AEA can sufficiently reduce the viscosity of cement paste and stabilize bubbles, due to the improved surface activity of cement particles, by adhering to the cement surface and enhancing hydrophobicity.

Several studies have shown that cement and surfactants (superplasticizer (SP) and other admixtures) strongly attract one another and that the adsorption of AEA increases the hydrophobicity of cement particles [10]. Various plasticizers can influence entrained air stability or viscosity of cement paste with AEA [11]. As pointed out in research [12], the viscosity of cement paste is the main factor in controlling foamed concrete’s stability and structure. Pastes with higher initial yield stresses resulted in higher stability of bubbles in the fresh state [12] because entrained air bubbles may serve as lubricants and attract cement particles to produce bubble bridges [13]. Another study demonstrates that SP’s presence reduces AEA’s efficiency because the system produces larger air bubbles [14,15]. The effect of SPs, AEA or their mixture on the cement hydration course is unclear. Some studies show that different types of AEA (with protein or synthetic nature) had different effects (behavior) on cement paste in the presence of a SP [16,17].

The effect of SP amount at a constant AEA level revealed that when the SP amount increases in the cement paste, the paste workability increases, but simultaneously, air content tends to decrease because air bubbles collapse [18,19]. Some studies explain that in the presence of SP, air content tends to reduce because of the negative charges present from the adsorption of SP on cement particles, which prevents the adsorption of AEA on the cement particles [20,21]. Although the interplay between AEAs and SPs is uncertain, mixtures with SPs mostly require higher AEA dosages to reach the desired air volume [13,22,23]. The adding sequence of AEA and SP and mixing intensity and time also influence the air content in the mix [19,24].

Our previous research [25] concluded that the AEA admixture, compared to different types of SPs, has the lowest influence on the initial setting time of cement paste, whereas SPs prolong the initial setting time by approximately 25%. When AEA and SPs were used together, the prolongation was significantly increased. In addition, it has been found that AEA significantly reduces the viscosity of cement paste. However, MWCNTs are hydrophobic, and their dispersion requires using SPs, commonly used in cementitious materials. Such SPs help to distribute MWCNTs evenly in the cement paste [26] and increase paste spread [27,28].

Despite the positive effect of AEA in reducing the density of lightweight foam concrete, the main disadvantage of such concrete is its meagre compressive strength, which makes its applicability insufficient. In order to expand the application area of such lightweight foam concrete, it is necessary to improve the compressive strength of the lightweight foam concrete. However, this is not a simple task, because, on average, the drop in compressive strength is 5.5% when 1% air is included in the mixture [15]. MWCNT additives are one of the ways suggested to enhance cementitious materials’ physical and mechanical characteristics [29,30]. Therefore, various fibers (polypropylene fibers, glass fibers, ceramic fibers, MWCNTs) are used to improve the compressive strength of such lightweight foam concrete [31,32]. However, the effect of polypropylene and glass fibers on foam concrete is insignificant.

On the contrary, it was found that MWCNTs can significantly improve the quality and strength of the walls of foam concrete pores and air voids, reduce the number of micropores and air voids, reduce their size (diameter), and ensure an even distribution of voids. Compared to MWCNT-free samples, in the samples with MWCNTs, a decrease in the average diameter of the pores was observed [33,34]. It was found that MWCNTs can improve the mechanical properties of foam concrete and reduce thermal conductivity due to the modified structure of the pore walls and a more even distribution of the pores by size. In addition, MWCNTs can act as bridges between nano-sized cracks or voids to increase crack resistance and ductility [12,35]. A study [36] investigated lightweight foam concretes without and with the addition of MWCNTs. According to the authors, MWCNTs have a filler function and participate in densifying the structure of the pore walls and, at the same time, reducing the size and volume of capillary pores and microvoids. Such a phenomenon manifests most prominently in the phase contact zone because MWCNTs can act as crystal nucleation sites due to the high surface energy, and hydration products are more actively deposited on their surface in the phase contact zone. That is why a larger (C–S–H) amount could be produced in the early hydration stage. Meanwhile, the binding ability of MWCNTs can be increased because of the increased functionalized surface of MWCNTs, which can create strong interfacial bonds with the cement matrix due to covalent bonds between functional groups and hydration products.

However, as many researchers indicated, adding MWCNTs reduces the spreadability and workability of cement mixtures [37,38,39]. Conducted studies [26,40,41] show that the spreadability of cement pastes depends on the amount of MWCNTs and the water/cement (W/C) ratio. By increasing the amount of MWCNT in the cement paste from 0.5 to 2.0% (of the cement content) and maintaining the W/C ratio of the cement paste at 0.4, 0.5, and 0.6, the spreadability results were as follows: as the W/C ratio increased, the spreadability increased, but when increasing the amount of MWCNTs in the cement paste, the spreadability decreased significantly. At a W/C ratio of 0.4, the spread of cement paste decreased from 60 to 40 mm; at a W/C ratio of 0.5, the spread decreased from 80 to 40 mm; and at a W/C ratio of 0.6, the spread decreased from 100 to 60 mm. Consistency studies of cement pastes with MWCNT suspension, SP, and AEA [42] showed that both additives significantly reduced the negative effect of MWCNTs on the consistency of cement paste.

Rheological studies [37] of cement mortars with different W/C ratios (0.4–0.5), amounts of MWCNTs (0–0.16% of cement content), and PCE (varying from 0.55 to 1.1% of the cement content) showed that the W/C ratio has the most significant influence on the mortar viscosity and surface tension, followed by the impact of the PCE content. The W/C and PCE ratios and the MWCNT content have the least influence. The effect of different amounts of MWCNTs (0.1 to 0.5% by weight of cement) and SP on the flowability of cement paste and the properties of hardened specimens investigated in the study [43]. It was found that the spread of samples with SP additive was higher than the analogous samples without SP additive. However, few studies have found the effects of MWCNTs and AEA on the foamed cement paste’s rheological properties and the influence on the stability of foam and entrained air. In a study [44], it was concluded that using MWCNTs in foam concrete can significantly increase the foam’s stability and the concrete’s compressive strength. This effect occurs because the foam concrete paste retains its strength longer, C_3_S hydration takes place more actively in the paste, and MWCNTs adhere very closely to the newly formatted C–S–H and ensure their growth. The addition of 0.05% MWCNTs (by weight of cement) to foam concrete increased the compressive strength of the specimens by about 70% (from 1.8 to 3.06 MPa) [45]. However, the stability of foam and its impact on the rheological properties of cement-based materials is still unclear and needs more in-depth analysis.

As we can see, the more admixtures and additives are used in cement paste, the more difficult it is to predict the rheological behavior of cement paste and the course of hydration without detailed research. Despite a large number of studies on the behavior of different SPs in cement paste, detailed research about the interaction of MWCNTs, AEA, and different types of SPs in cement paste is still unclear and requires further study. That is why, to develop MWCNTs in lightweight foamed concrete, it is important to research how AEA interacts with MWCNTs and different types of SPs and how this interaction influences foam stability.

The same understanding of how MWCNTs, AEA, and SPs of different types affect the cement paste’s electrical conductivity and influence rheological properties such as the spread and setting time of foamed cement pastes can bring necessary knowledge for the successful use of such admixtures in concrete technologies. Thus, reasoned recommendations and further research are highly relevant before choosing the appropriate amount of MWCNTs to include in paste with AEA. The research results obtained using widespread methods are highly relevant before selecting the amount of MWCNTs, and types of SP and AEA in the lightweight concrete compositions.

## 2. Materials and Methods

### 2.1. Materials

Ordinary Portland cement (OPC) CEM I 42.5 R produced by JSC “Akmenės cementas” (Akmenė, Lithuania) following standard EN 197-1 [46] requirements was used for the tests. The OPC had a specific surface of 4200 cm^2^/g, bulk density of 1.1 g/cm^3^, initial setting time of 140 min, final setting time of 190 min, and alkali content up to 0.8%. Mineral composition in percentages: C_3_S—56.64, C_2_S—16.72, C_3_A—8.96, and C_4_AF—10.59. OPC particle size ranged from 1 to 100 μm, and 50% of OPC particles were 15–30 μm.

The pellets, named GRAPHISTRENGTH CW2-45, containing multi-walled MWCNTs with purity >90% at a concentration of 45% (by weight of mixture), dispersed in carboxymethylcellulose at content of 55% (by weight of mixture), provided by a company ARKEMA (Colombes, France) were used.

AEA, manufactured by “UFAPORE TCO” (Fredrikstad, Norway), is a white powder produced on the sulfonate base of sodium alkenes. It ensures the formation of small pores in the cement matrix. The AEA content in the cement matrix recommended by the manufacturer is 0.01–0.06% of the mass of the binder. Research [47,48] shows that the most appropriate content of AEA to be used for tests (upon striving to avoid deterioration of the strength properties) should be 0.03% [25].

The following three different types of SP were used in the research: polymer-based synthetic polycarboxylate ester SP(PCE), polyacrylate-based SP(PA) manufactured by BASF (Trostberg, Germany), and modified lignosulphonate SP(LS) manufactured by STACHEMA LLC (Kolín, Czech Republic) plasticizer. SPs were used in a liquid state. The active substance content of SP(PCE) in water solution was 27%, SP(PA) content was 27%, and SP(LS) was 31%. The characteristics of SPs, as received from the producer, are presented in Table 1. Our earlier research [49] concluded that the most appropriate amount of SPs in pastes with MWCNTs addition is 0.2% of BWOC. However, the amounts for SPs were constant in the mixtures (0.2% of the cement content) and recalculated according to the concentration of a dry substance in the solution of each SP. To evaluate how 0.2% of SPs and AEA will affect EC and pH values of distilled water solution (100 g), measurements of EC and pH of prepared solutions were performed at 20 °C (Table 1).

### 2.2. Preparation of Suspensions and Cement Paste

#### 2.2.1. Preparation of MWCNTs Water Suspensions

For preparing water suspension compositions (Table 2), the pellets containing the necessary amounts of pure MWCNTs—0.0, 0.004, 0.04, 0.4, 4.0, and 40 g—were chosen. These amounts were chosen considering that half of the weight in the pellets consists of carboxymethylcellulose. Furthermore, this amount corresponds to the percentage contents of cement (0.00005, 0.0005, 0.005, 0.05, and 0.5%) of the compositions presented in Table 2. Such amounts of MWCNTs were selected based on the experience of other authors [50,51,52]. Weighted pellets were immersed in 100 mL of hot (95–100 °C) distilled water for 10 min without mixing. Afterwards, pellets were subjected to ultrasonic treatment by the ultrasonic disperser UZDN-2T (frequency 22 kHz, power 480 W) for 5 min in a 200 mL capacity cylinder. Prepared MWCNT suspensions were diluted with 980 g of distilled water (this quantity was calculated according to the cement paste composition W/C ratio of 0.27, and the final amount resulted in 1080 g) and mixed with a laboratory mixer with a vertical rotation axis by forced mixing with a speed of 125 rpm for 2 min. Experimental water suspensions with MWCNTs were cooled to room temperature (20 °C), and then dynamic viscosity of the control sample and all 5 MWCNTs amounts were measured.

#### 2.2.2. Preparation of Fresh Suspensions for Foam Index Test

The foam index test aims to anticipate the compatibility of the combinations of AEA–MWCNTs, AEA–SPs, AEA–MWCNTs, and AEA–MWCNTs–SPs—first without cement and then with cement. The foam index test procedure was conducted for compatibility with AEA–OPC–MWCNTs, AEA–OPC–SPs, AEA–OPC–MWCNTs, and AEA–OPC–MWCNTs–SPs. The test followed the methods presented in [53]. For use in a test of just AEA, a solution was made with 100 mL distilled water and 0.15 g AEA. Then, 20 mL of prepared AEA solution and 20 mL water were mixed in a 100 mL cylinder. The same 20 mL of AEA solution was used for SPs, but 0.07 g SP was dissolved in the 20 mL water. In the case of MWCNTs, a 20 mL AEA solution was used. An volume of 20 mL of water was prepared with different amounts of MWCNTs (from 0.000037 to 0.37 g). When AEA, SPs, and MWCNTs were tested together, 0.07 g of SP and a specific amount of MWCNTs (from 0.000037 to 0.37 g) were added to 20 mL of water and 20 mL of the AEA solution was used. 

The influence of cement was tested for the mentioned components. For this purpose, 8 g of cement was used for each suspension mentioned above. The compositions of the tested suspensions are presented in Table 2.

#### 2.2.3. Preparation of Fresh Paste

Also, for preparing fresh cement pastes with MWCNTs only and MWCNTs and AEA (Table 3), the same prepared water suspensions with MWCNTs and MWCNTs with AEA were used. For this purpose, pure K-C0 and five paste compositions with different amounts of MWCNTs were studied. The same was true for control paste K-C0-A, and five paste compositions with different amounts of MWCNTs and AEA were prepared. The water to-cement ratio (W/C) in all pastes was the same (0.27). The amount of MWCNTs in the forming mixture varied from 0.00005 to 0.5% (by weight of cement), and the amount of AEA was constant at 0.03% (by weight of cement). Weighted AEA was taken up in 1080 g suspension with the selected amount of MWCNTs already present. Fresh cement pastes were mixed with a laboratory mixer with a vertical rotation axis by forced mixing at 125 rpm for 4 min.

### 2.3. Methods of Testing

#### 2.3.1. Foam Index Test

Two 100 mL graduated cylinders were filled with prepared suspension, and 8 g of cement was added to one of them and shaken continuously for 30 s. After shaking, the initial volume of the foam height was registered immediately and after 5 min at rest. This procedure was repeated twice for each sample group, and the reported foam volumes corresponded to the average value. The absolute volume of the foam and the relative lack of change between its final and initial volumes indicate the foam’s stability. The relative change in the foam stability between the surfactant solution and surfactant solution with cement can imply the compatibility between the cement and each surfactant.

#### 2.3.2. Viscosity Measurements

The effects of different SPs, MWCNTs, and AEA on the dynamic viscosity of fresh suspension were tested using a vibro-viscometer SV-10 (capacity up to 12.000 mPa·s, accuracy 0.01 mPa·s). The dynamic viscosity of fresh suspension was measured immediately after the preparation.

#### 2.3.3. Electrical Conductivity and pH Testing

The effect of different SPs on the values of EC and pH of the water solution and cement pastes with MWCNTs, AEA, and different SPs were performed with the MPC 227 instrument manufactured by Mettler Toledo (pH sensor InLab 410, measuring accuracy 0.01; EC sensor InLab 730, measuring range 0 mS/cm–1000 mS/cm). All measurements were performed at a temperature of 20 °C. Electrical conductivity is a state measured by applying an electrical field between two electrodes (+ and −) that initiate electric current. The Mettler Toledo device conductivity sensors are 2-in-1, i.e., both polarities are integrated into one housing. In the case that 4 ring sensors are used, two rings are used for voltage engagement and the other two for current, which is proportional to ionic strength in the solution. For engagement, AC (alternating current) voltage is used for a reason: the “plating” effect (ions gathering at conductivity plates and staying there as immobile particles, thus decreasing the area of the plates, i.e., conductivity cell changes). Further signal processing runs with conversion from AC for data output.

#### 2.3.4. Setting Times

Setting times of cement paste with different amounts of MWCNTs and admixtures were determined based on standard test methods by EN 196-3 [54]. The tests were performed with a needle penetrating every 10 min. The tests were carried out at a temperature of 20 °C.

## 3. Results

### 3.1. The Foam Index Test

**Figure 1:**
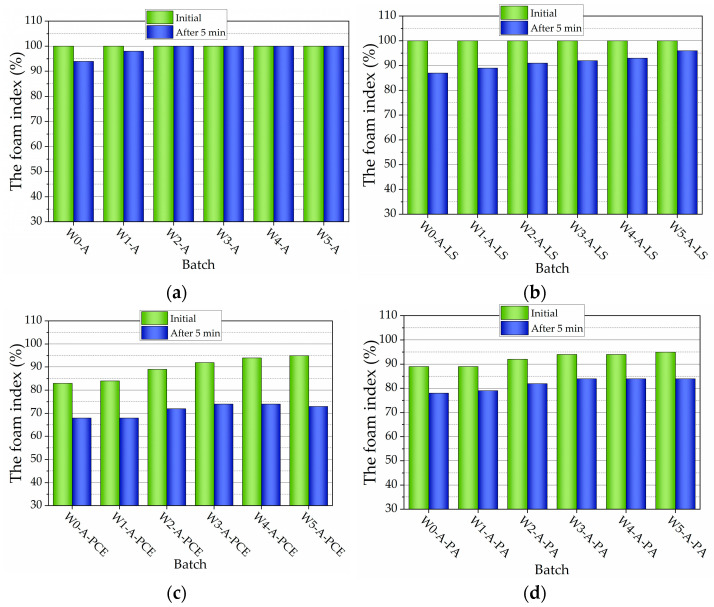
Foam index test results of AEA with (**a**) different amounts of MWCNTs, (**b**) different amounts of MWCNTs and LS, (**c**) different amounts of MWCNTs and PCE, and (**d**) different amounts of MWCNTs and PA.

**Figure 2:**
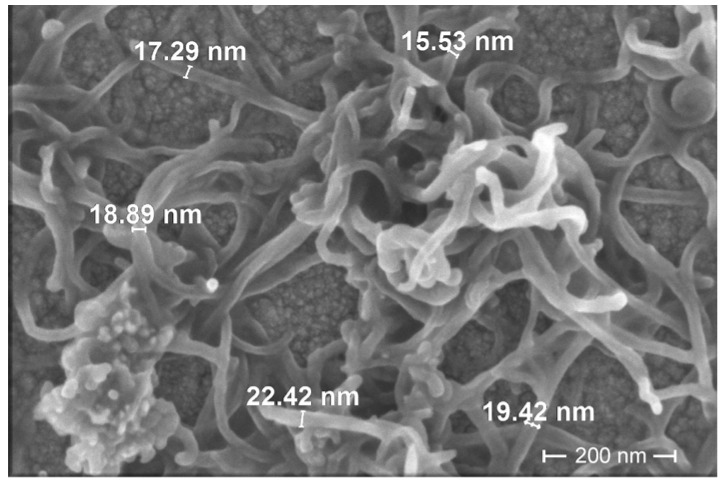
MWCNT filaments dispersed in water.

The foam index test is proposed in many studies [2,55,56,57], but currently, there is a lack of standards for the establishment of foam index with and without the effect of cement. Foam index test Figure 1a presents the test results expressed as the percentage of occupied volume of the graduated cylinder by the foam. From these, foam stability can be implied by the lack of change in occupied volume between 0 and 5 min. By evaluating the occupied volume, after 5 min, MWCNT-free composition shows a slight decrease (by 6%) in occupied volume. Compositions with 0.00037–0.37 g of MWCNTs showed no change in volume [58]. The cement paste inside the foam structure may behave differently than the unfoamed reference cement paste. MWCNTs seem to behave as dense matter, reinforcing the bubble walls [59]. This premise is confirmed by the image of MWCNT filaments dispersed in water (Figure 2). The structure of the filaments suggest that air bubbles can be caught in the filaments network.

This assumption is proven in a study [60]. This study tested the rheological behavior of complex foam and foam with small-bead suspension. It was concluded that complex foam is more fluid than foam with small-bead suspension. It was concluded that small-bead particles packed in the foam structure behave as dense granular matter, exhibit particle size effects, and increase foam stability.

The presence of SP(LS) (Figure 1b) does not decrease the initial volume of foam. When LS and MWCNTs are used together, the same initial volume of foams is not decreased. At a time of 5 min after shaking, the most significant volume change belongs in compositions with the lowest CNT amounts. The presence of SP(PCE) significantly decreased the initial volume of foam, by 17% (Figure 1c). An increase in MWCNT amount improves foam stability and increases the initial volume of foam. However, after 5 min, the loss of foam volume reaches 20%. When SP(PA) is used (Figure 1d), the initial volume of foam decreases by 11%. An increase in MWCNT amount improves foam stability. After 5 min, the loss of foam volume reached 11%.

This research shows that SP(PCE) and SP(PA) decrease the initial volume of foam, and after 5 min, the most significant volume change belongs to PCE. It is known that SPs impede air intake in the composition [1], but mainly, it is observed for SP(PA) and SP(PCE) superplasticizers. SP(LS) superplasticizer shows less change in volume.

**Figure 3:**
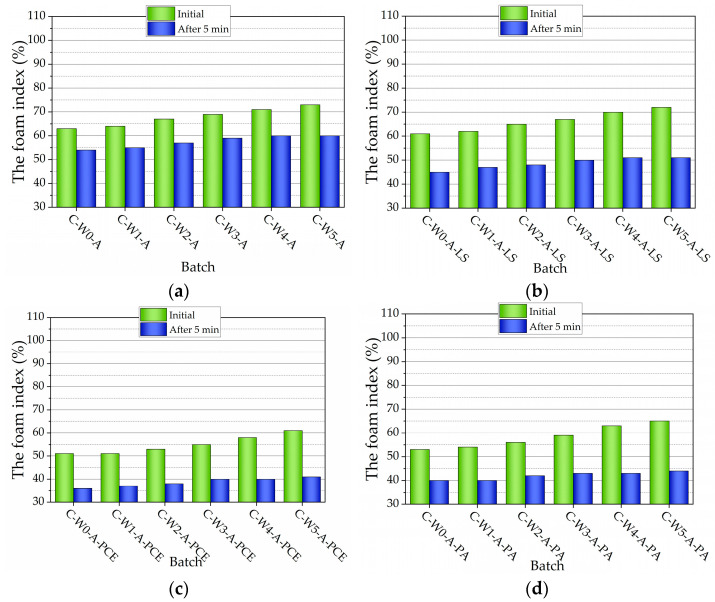
Foam index test results of AEA with cement and: (**a**) different amounts of MWCNTs, (**b**) different amounts of MWCNTs and LS, (**c**) different amounts of MWCNTs and PCE, and (**d**) different amounts of MWCNTs and PA.

The addition of cement decreased the initial volume of AEA foam (Figure 3a) by 37%, compared to pure AEA foam. An increase in MWCNTs increases the initial volume of AEA foam. However, the initial volume decreased by 36–27% compared to the same compositions without cement. After 5 min, the loss of foam volume reached 40%, compared to the same foam composition without cement.

The presence of SP(LS) and cement (Figure 3b) decreased the initial volume of foam even more, (to 39%) compared to pure AEA foam. With the increase in the amount of MWCNTs, the initial volume of AEA foam increases. Compared to the same compositions without cement, the decrease in initial volume reached 38–28%. After 5 min, the loss of foam volume reached 49% compared to pure AEA foam, and in the range of 47.2–45.6% compared to the same foam composition with MWCNTs and without cement.

The presence of SP(PCE) (Figure 3c) significantly (by 49%) decreased the initial volume of AEA foam. An increase in MWCNT amount improves foam stability, and initial volume decreases by 39.3–35.8% compared to the same compositions without cement. After 5 min, the loss of foam volume reached 56.6%, compared to pure AEA foam, and in the range of 56–57% compared to the same foam composition with MWCNTs and without cement.

When SP(PA) is used (Figure 3d), the initial volume of foam decreases by 47%. Increasing the amount of MWCNTs improves foam stability, and initial volume decreases by 39–31.6%. After 5 min, the loss of foam volume reached 48–50%, compared to the same foam composition without cement. A higher amount of SPs can cause some thixotropy effect in cement paste [61] when the attraction forces between cement particles are reduced, due to steric repulsion. As a result, the colloidal network is not expected to be stronger or to form faster in the case of deflocculated pastes.

The addition of cement decreased the AEA foam stability. The foaming ability of AEA is affected by the presence of calcium ions, and AEA contributes to the formation of an inorganic shell around the air voids [62,63]. It happens either due to adsorption onto solid surfaces or due to the interaction of this anionic AEA with the high concentration of calcium ions (Ca^2+^) present in the solution, thus reducing the amount of free AEA in the solution. The presence of calcium ions increases surface tension for anionic surfactants since the calcium ions coagulate anionic surfactants and reduce the number of surfactants free for interface adsorption [8,57,64,65,66].

It can be concluded that MWCNT addition works as the matter to improve foam stability. However, the stability of foams greatly depends on the used SPs. SP(LS) contributes to foam stability the most and SP(PCE) contributes to a lesser extent.

### 3.2. Dynamic Viscosity of Foam

**Figure 4:**
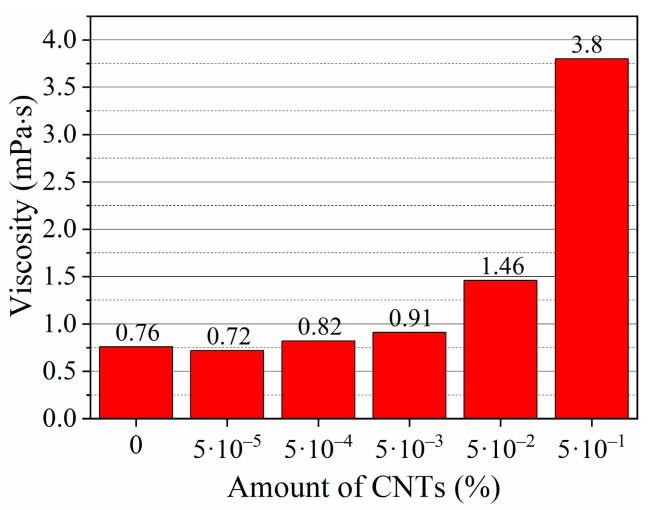
The dynamic viscosity of pure water and water suspension with different amounts of MWCNTs.

To clarify how AEA admixture can interact with MWCNTs, cement, and SPs, viscosity testing of the abovementioned suspensions without cement and suspensions with cement was conducted. The dynamic viscosity of compositions was tested the same as in the foam index test, immediately after mixing and after 5 min. For comparison, distilled water and MWCNT water suspensions of different concentrations were also tested (Figure 4). In some studies [67,68,69], it was pointed out that MWCNTs can affect the viscosity of a resulting suspension. When the smallest amount of MWCNTs (0.000037 g) was investigated, the results showed that viscosity slightly decreased to 0.72 mPa·s, compared to the viscosity of pure water (0.76 MPa·s). However, when the amount of MWCNTs in the suspension increases from 0.00037 to 0.37 g, the dynamic viscosity sharply increases and reaches 3.8 mPa·s. This means that the highest amount of MWCNTs, 0.37 g, increases suspension viscosity by five times compared to pure water viscosity.

**Figure 5:**
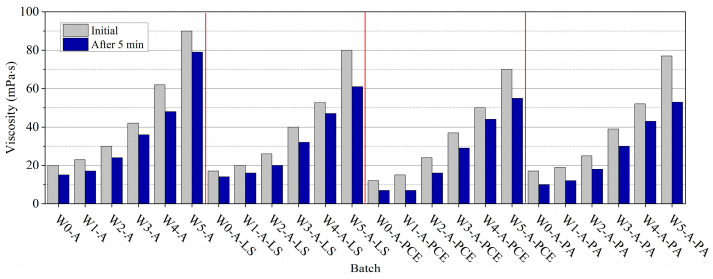
The dynamic viscosity of foamed AEA with MWCNTs and SP(LS), SP(PCE), and SP(PE) after different times of measurement.

The interaction of AEA and MWCNTs in the suspensions was studied. Immediately after mixing the smallest amount of MWCNTs (0.000037 g), the sample’s viscosity slightly increased [4] (about 13%) in comparison to the control MWCNT-free sample (Figure 5). When samples with higher amounts of MWCNTs (0.00037–0.37 g) were tested, the viscosity increased in the range 30–90 mPa·s, reaching 4.5 times compared to the control MWCNT-free sample. Observations of how the stability of the foam changes over time shows that after 5 min, the viscosity mainly decreases in the control MWCNT-free sample and in the samples with the lowest (0.000037 and 0.00037 g) MWCNT amounts (in the range 26–20%), compared to viscosity values right after mixing. In the sample with the highest amount of MWCNTs, the viscosity reduction was not high—only about 12%.

The same trend is observed when SPs are added to the composition (Figure 5). The only difference is that SP(LS) reduces the viscosity less (by 15–13%, compared to samples without SP(LS) addition). The SP(PCE) is characterized by the most visible reduction of viscosity of the samples. SP(PCE) reduces the viscosity of the samples by 40–23%. SP(PA) reduces viscosity by 15–16%. After 5 min, the viscosity mainly decreases in samples with SP(PCE) addition. It is noticeable that higher viscosity changes are closely related to the air-bubble amount and the stability in foamed suspension [56,57]. This means that in foamed suspensions, with an increase in MWCNT amount, a significant number of air bubbles are trapped, and MWCNT filaments prevent bubbles from escaping, and as a result, the viscosity significantly increases.

The presented results conclude that the higher the amount of MWCNTs used, the higher the sample’s viscosity. When admixtures of AEA and MWCNTs are used, their synergistic effect on viscosity is noticeable. If MWCNTs are used exclusively, the viscosity of the suspension increases to 3.8 mPa·s. When AEA is used exclusively, the viscosity of the suspension increases to 20.1 mPa·s. When AEA and MWCNTs act together, the viscosity rises to 90 mPa·s.

### 3.3. Dynamic Viscosity of Foamed Suspension with Cement Addition

**Figure 6:**
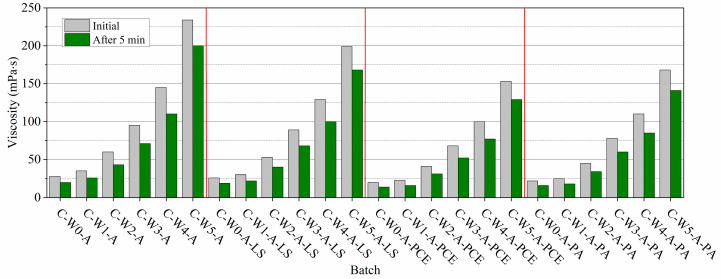
The dynamic viscosity of foamed AEA with cement, MWCNTs and SP(LS), SP(PCE), and SP(PE) after different times of measurement.

The effect of cement addition to the foam with different amounts of MWCNTs was investigated (Figure 6). In the presence of cement, higher amounts of MWCNTs (0.0037–0.37 g) significantly increase the viscosity of the samples up to 234 mPa·s, which is a 2.6 times increase compared to the control cement-free sample. Observations of how the stability of the foam changes over time show that after 5 min the viscosity mostly decreases in the control MWCNT-free sample (to 29%); in the sample with the highest amount of MWCNTs, the reduction of the viscosity was about 15%. The addition of cement in the foam with SP(LS), SP(PCE), and SP(PA) increases viscosity by 34.6, 40, and 22.7%, respectively, compared to the control cement-free sample (Figure 5). With the increase in MWCNT amount in suspensions, viscosity increases up to 2.5, 2.1, and 2.2 times, respectively. After 5 min, the viscosity decreases to 15.6%, 15.7%, and 16.1%, respectively. MWCNTs in suspension can exhibit solid particle size effects [60]. In our case, MWCNT filaments are solid particles and interact with all suspension components. When cement is added to suspension, yield stress controlling foam flow properties increases. As reported, foams made with small solid particles reveal granular packings confined between foam bubbles and the resulting yield stress increased. We can conclude that the addition of cement increased foamed AEA viscosity but decreased foam stability because of the interaction of anionic AEA with the (Ca^2+^) ions present in the solution, thus reducing the amount of free AEA in the solution [62]. Another reason [8,65,66,70] is that (Ca^2+^) ions increase surface tension for AEA since the (Ca^2+^) ions coagulate anionic surfactants and reduce the number of surfactants free for interface adsorption.

### 3.4. Properties of Fresh Cement Paste

#### 3.4.1. Electrical Conductivity

**Figure 7:**
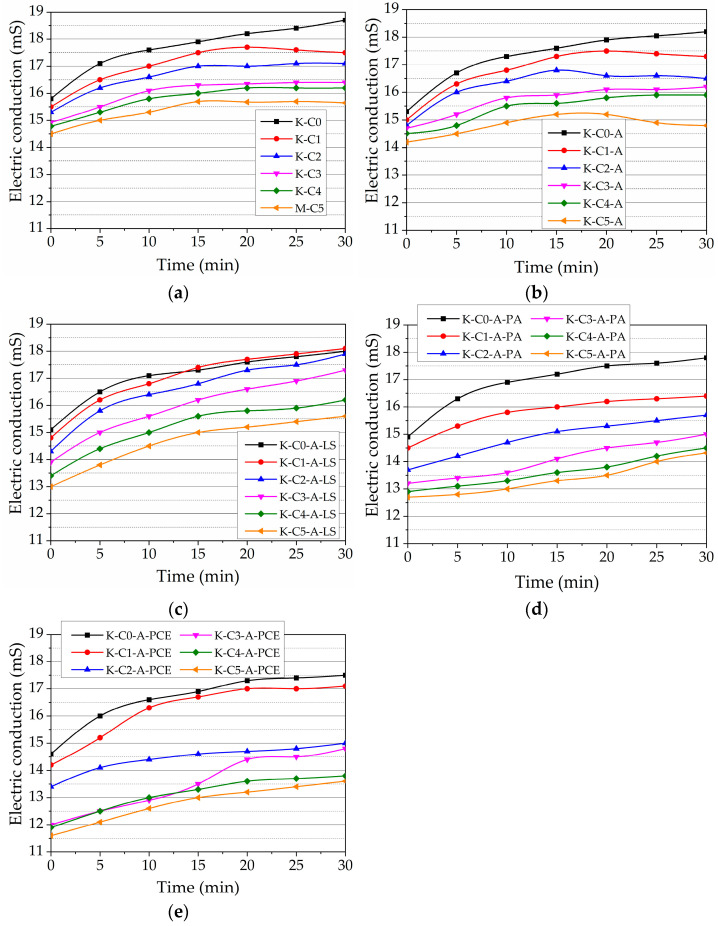
The EC of cement paste: (**a**) with different amounts of MWCNTs, (**b**) with different amounts of MWCNTs and AEA, (**c**) with different amounts of MWCNTs and AEA, and SP(LS), (**d**) with different amounts of MWCNTs and AEA, and SP(PA), (**e**) with different amounts of MWCNTs and AEA, and SP(PCE).

In order to better understand the fresh properties of cement paste and the impact of different amounts of MWCNTs in cement paste, electrical conductivity measurements were performed. The measurements were performed within 30 min because the significant and rapid increase in electrical conductivity during this period describes well the transition of cement mineral ions into solution [71]. The results show (Figure 7a) that as the amount of MWCNTs increases, the initial EC values of cement paste decrease. Considering that C_3_A and ferritic phase minerals enter the solution first, it can be assumed that their dissolution is slowed the most with an increase in the MWCNT amount. It is thought that the MWCNTs’ impact determines the MWCNT makeup—MWCNT filaments and separate tubes wrap around the surfaces of the cement particles and block the cement mineral transition into solution [49]. The EC in the control sample paste was 15.8 S/m. After increasing the amount of MWCNTs in the cement paste from 0.00005 to 0.5%, EC values decrease from 15.5 S/m to 14.5 S/m. The smaller amounts of MWCNTs (0.00005–0.0005%) decrease EC values by approx. 3%; higher MWCNT amounts decrease EC by 8.2%. It can also be assumed that the carboxymethylcellulose contained in MWCNT pellets as a binder can also affect EC values at the beginning of hydration because carboxyl-methyl cellulose is a weak acid, able to reduce the cement ions’ transition into solution [72].

Further research showed that the smaller the amounts of MWCNTs used in cement paste, the higher the electrical conductivity values achieved after 30 min. The difference in EC values between the control paste and the paste with the highest amount of MWCNTs was 3.35 S/m (approximately 18.5%). These results show that MWCNTs influenced the dissolution of cement minerals and, therefore retarded the hydration process.

Low EC and alkaline pH in solution characterize AEA. When AEA is used in the control paste (Figure 7b), the EC values in the pastes decrease by about 3.2%, but in the paste with the highest amount of MWCNTs, EC decreases by about 2.1%. This shows that AEA has very little influence on the cement dissolution process. At the finish of measurements, the EC values in control paste K-C0-A were 2.6% lower than in pure paste K-C0. With an increase in MWCNT amount in the paste, EC values decrease to 5.1%. This means that AEA, characterized by low EC and alkaline pH in solution, has little influence on cement dissolution [25].

Next, the impact of equal amounts of SP(LS), SP(PA), and SP(PCE) on the paste’s EC with AEA and different amounts of MWCNTs was tested. A satisfactory amount of SPs for use in the paste was established in a study [73]. The interaction between AEA and SP in the cement paste can be affected by several factors, such as the SP architecture, side-chain length and charge density, the chemical and grain-size composition of OPC, and environmental conditions [74]. In the presence of an SP, different AEA types (protein or synthetic) exhibited distinct impacts on cement paste [75]. However, different AEA types (with protein or synthetic nature) had different effects on cement paste in the presence of an SP [75]. The studies on using AEA and SPs together are not abundant, and the effect of AEA on cement hydration when SP is used concomitantly is unclear. According to some studies, a combination of AEA and various SPs can influence the aeration process [76]. However, the involvement of MWCNTs in interactions with AEA and SPs has not been sufficiently investigated.

SP(LS) has the greatest EC values in the solution (Table 1), which explains why the EC values in the cement paste are relatively high (Figure 7c). The control MWCNT-free paste’s EC value is 15.3 mS. As seen, the initial EC values in paste depend on the amount of MWCNTs in the composition—with an increasing amount of MWCNTs, the initial value of EC in pastes decreases from 14.8 mS to 13 mS. According to [77], SP(LS) might have a high EC due to the high number of R-SO^−3^ groups initially present in sulphonates. With increasing amounts of MWCNTs, the EC values in pastes decrease by 1.98%, 5.3%, 7.9%, 11.26%, and 13.9%, respectively, compared to the control C0-A-LS paste. During the first 15–20 min, the ECs of all composition pastes increase in the range of 14.5–17.0%, and at the finish of measurement, the EC values of the control and pastes with 0.00005–0.005% of MWCNTs are practically the same. The ECs of pastes with higher amounts of MWCNTs are 7.6% and 12.6% lower than in the control paste. Relatively high EC values in the paste with SP(LS) can be explained by the AEA’s sulfonyl groups’ influence, which can promote faster dissolution of cement minerals and may prevent the reaction between the sulphonate group of SP(LS) and C_3_A mineral. When the cement minerals’ ions are passed into the solution, they can fast react with the sulphonate groups of SP(LS), and as a result, the formation of ettringite occurs [78], and as a consequence, a significant EC reduction is observed in the paste.

The influence of SP(PA) on the EC values of pastes is presented in Figure 7d. SP(PA) does possess low EC values in the solution (Table 1), which is why EC values in the cement paste are low. The influence of SP(PA) on the EC values of the control sample is tiny, and the EC values reach 14.9 mS. In comparison to K-C0-A without SP (Figure 7b), EC values decreased by 2.6%. With an increase in MWCNT amount in the paste, the previously observed tendencies remain—the initial EC values in paste decrease by 2.7, 8.1, 11.4, 13.4, and 14.8%, respectively, compared to control paste K-C0-A-PA. Compared to compositions with SP(LS), it can be pointed out that in the pastes with SP(PA), a decrease in initial EC values is more expressed. It can be observed that the highest decrease in EC is observed in pastes with lower amounts of MW. In the pastes with higher amounts of MWCNTs (0.05 and 0.5%), the change in EC is small. At the finish of measurements, the EC values of the control and pastes with 0.00005–0.005% of MWCNTs significantly differ and reach 17.9, 16.4, 15.8, and 15.2 mS, respectively. The ECs of pastes with higher MWCNTs amounts differ slightly and reach 14.9 and 14.7 mS.

Further, the impact of SP(PCE) on the pastes’ EC values was tested (Figure 7e). By using this SP, the EC values decreased even more. The control paste K-C0-A-PCE’s initial EC value is 4.6% lower than in K-C0-A SP-free paste’s (Figure 7b) EC values. Compared to the control paste K-C0-A-PCE, the initial EC values decrease by 2.7, 8.22, 16.44, 18.5, and 20.6%, respectively, with increasing MWCNT amount in the paste. It can be pointed out that, in comparison to the previously described SP(PA), a decrease in initial EC values is even more noticeable. It is observed that SP(PCE) effectiveness is more pronounced in pastes with higher amounts of MWCNTs. At the finish of measurement, the EC values of control K-C0-A-PCE and pastes with 0.00005% of MWCNTs are very similar, but EC values of pastes with higher amounts of MWCNTs differ significantly and are 14.2, 13.1, 20.5, and 21.6% lower, respectively, than in pointed control paste. Such results can be explained by the fact that the EC values of SP(PCE) mainly depend on the number of carboxyl groups COO^−^ and differences in SP(PCE) architecture [79]. Generally, it can be concluded that all tested MWCNTs, AEA, and SP admixtures in cement paste retarded dissolution and decreased the paste’s EC. The largest impact on EC decrease within 30 min resulted from the highest MWCNT amount—an EC decrease up to 18.5%. When MWCNTs, AEA, and SP admixtures are used together, due to their interaction, the impact of used admixtures in lowering EC increases up to 28–29%, compared to the pure control admixture-free cement paste’s EC values.

#### 3.4.2. Spread Area

**Figure 8:**
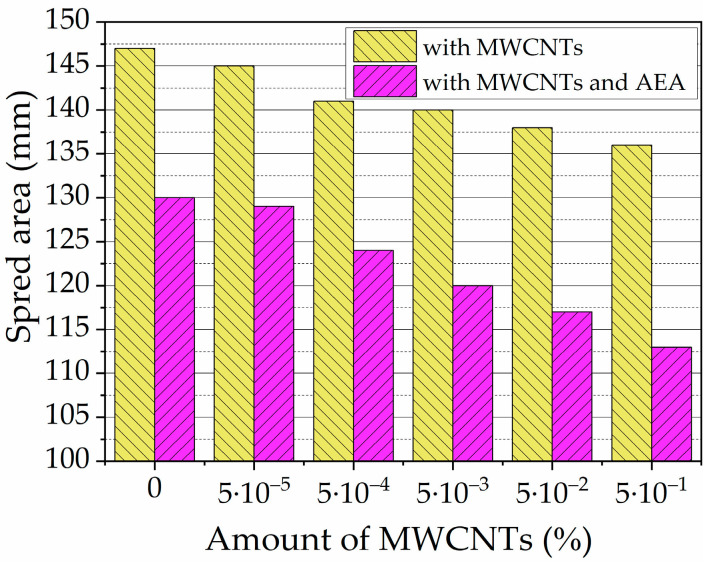
Spread area, in mm, of the OPC paste with MWCNTs and in the same paste with AEA admixtures.

Increasing the air content decreases the viscosity of concrete and thus enhances workability. An increase in workability by adding AEA in concrete is also reported by Hammad Ahmed Shah et al. [13]. Figure 8 shows the result of the spread test of cement pastes with different amounts of MWCNTs and cement pastes with different amounts of MWCNTs and AEA. The presence of MWCNTs and the presence of AEA decrease the dissolution of cement minerals, and thus, the viscosity of the paste decreases. Results show that with increased amounts of MWCNTs, 0.00005–0.005% paste’s spread values decrease to 7.5% compared to MWCNT-free K-C0 control paste’s spread. As presented in Figure 2, MWCNT filament structure can decrease paste flowability.

According to [39], all carbon-based nano-fillers, including MWCNTs, decrease the workability of a cement paste. The higher the amount of MWCNTs used, the lower the paste spread [41]. The authors revealed that adding MWCNTs in a dosage of (0–0.1% BWOC) leads to decreased flowability. The same tendencies were observed in a study [26], where the mini-slump test of ordinary cement pastes with different W/C ratios of 0.5, modified by the different dosages of MWCNTs, was performed. The spreading diameter of cement paste decreased by 14.5% with the addition of 0.5% BWOC of MWCNTs. Researchers Li and Lin [80] observed the decrease in flowability of magnesium phosphate cement modified by MWCNTs and explained the observed changes by bridging, pore-filling, and nano-size effects of MWCNTs in the cement matrix.

The spread values significantly decreased in the paste using both MWCNTs and AEA . The spread value of control paste K-C0-A is 11.6% lower than control paste K-C0 without any admixtures. In the presence of AEA, the pastes’ spread values decrease significantly compared to pastes without AEA, to 15.25%, when MWCNT amount increases from 0.00005 to 0.5% in the paste. The AEA admixture, which created an air-bubble structure, limits the paste’s mobility, as seen from the spread results of K-C0 and K-C0-A pastes. Trapped MWCNT filaments created an air-bubble structure and further reduced the spread of the paste.

**Figure 9:**
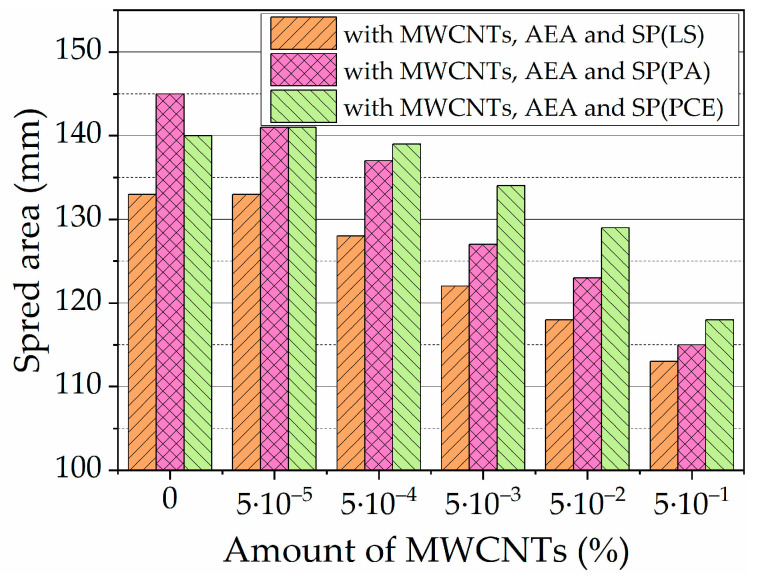
Spread area, in mm, of the OPC paste with AEA, and different amounts of MWCNTs and SPs.

The results of spread values of pastes with different SPs are presented in Figure 9. The presence of SP(LS) showed a little-improved spread value in paste with AEA (by 0.8–3.8%) when the MWCNT amount in the paste increased to 0.05%. With the highest amount of MWCNTs, no improvement is observed in the spread values of the paste, compared to the spread of control pastes with MWCNTs and AEA admixture, presented in Figure 8. It is evident that, compared to the spread of pastes with MWCNTs and AEA admixture, SP(LS) improve the fluidity of pastes very little. This can be explained by higher EC values in the cement paste when SP(LS) is used. When SP(PA) and SP(PCE) were used, the EC values in cement paste were lower. Such results can be explained based on our presented hypothesis [71] that the nature of SP(LS) provides early ettringite formation. In the case of MWCNTs in the paste, an additional effect occurs—MWCNT filaments trap air bubbles and interact with the earliest formed ettringite needles’ structure to decrease the spread of the paste.

When SP(PCE) was used, the spread values for visible pastes increased compared to the same paste with SP(LS) values. In the control paste and pastes with lower amounts of MWCNTs (0.00005–0.05%), the spread values are 8.5–10.5% higher than in the same composition pastes with MWCNTs and AEA admixture. However, when the MWCNT amount is the highest, the spread values have improved by 5.8% only.

The influence of SP(PA) on the spread values of pastes shows that SP(PA) efficacy is a little lower than SP(PCE). SP(PA) spread values in the control paste are 11.2% lower than in pastes with MWCNTs and AEA admixture. In the pastes with lower amounts of MWCNTs (0.00005–0.0005%), the spread values are in the range of 8.5–8.8% higher, but in the pastes with higher amounts of MWCNTs (0.005–0.5%) the spread values are only 5.5–2.6% higher than in the same composition pastes with MWCNTs and AEA admixture.

Finally, it can be concluded that the pastes’ EC results support the pastes’ spread results. SPs with higher cement-paste EC reduction possibilities (the most visible are SP(PCE) and then SP(PA)) more efficiently increase spread values in pastes with AEA and MWCNTs, and those with lower EC reduction possibilities (SP(LS)), change the paste EC values the least.

### 3.5. Setting Time of Fresh Cement Paste

**Figure 10:**
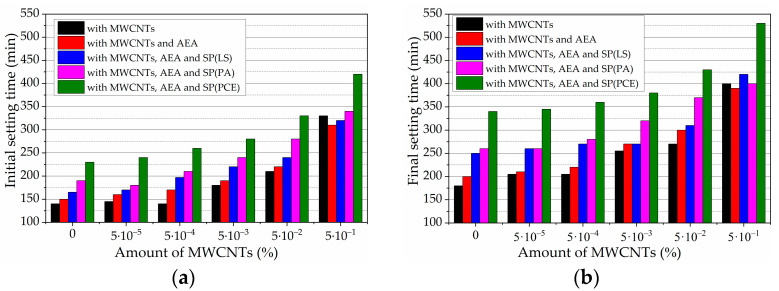
(**a**) Initial setting times and (**b**) final setting times of cement pastes with AEA, various SPs, and different amounts of MWCNTs.

The results of initial setting times of fresh cement pastes with different amounts of MWCNTs and all used admixtures (AEA admixture and different types of SP) are presented in Figure 10. In the pastes with MWCNTs only, the lower amounts (up to 0.005%) prolong the initial setting time by 22%, but higher amounts (0.05 and 0.5%) prolong the setting time by 1.5 and 2.4 times, respectively. Due to decreased cement dissolution and lower EC values, the Ca ions pass much more slowly into the solution, and the setting time is prolonged. In our earlier research [73], the retardation of initial and final setting times in the case of a modification of cement paste by MWCNTs in the dosage of 0.005–0.5% BWOC was by about a factor of two. Research results showed that MWCNT amounts above 0.005% may significantly retard the hydration of cement minerals [49]. This is despite some researchers pointing out [81,82] that higher amounts of MWCNTs (0.05–0.5%) differently influence the initial setting time, because different admixtures employed for MWCNT production can be used.

Compared to the control MWCNT-free paste, the AEA admixture, characterized by the lowest electrical conductivity in water solution, significantly prolongs the initial setting time, by about 7%. With the increase in MWCNT amount up to 0.0005% in the paste, initial setting time increases up to 17.6%, but when the MWCNT amount is higher, initial setting time increases only up to 4.5% and with the highest MWCNT amount, initial setting time decreases by 7.1%.

SPs show different impacts on the initial setting times of pastes. When the MWCNT amount increases to 0.005% in the paste, tested pastes with SP(LS) prolonged initial setting time by up to 1.46 times; SP(PA) and SP(PCE) required almost 1.6 and 1.73 times longer times to start setting, in comparison to control paste with MWCNTs and AEA admixture. When the MWCNT amount is the highest (0.5%) SP(LS) prolongs the initial setting time by 2.0 times, and SP(PA) and SP(PCE)prolong the initial setting time by 2.2 and 2.8 times, respectively. It should be mentioned that, depending on the MWCNT amount in the paste, the setting time can be extended from 1.46 to 1.73 times when the MWCNT amount is not higher than 0.005% and to 2–2.8 times when the MWCNT amount is highest. Setting time is dependent on SPs’ pH, the electrical conductivity values in solution, and the values of EC in the cement paste. As can be seen from Table 1, the number of ions in different SP solutions varies considerably. Similar values of EC of SPs are also reported in [17]. The results show that, between all tested SPs, SP(LS) causes the least retardation of the initial setting time and the final setting time, both used individually and in combination with AEA and MWCNTs.

The result that SP(PCE) shows the most increased initial setting time can be explained by the fact that the AEA and SP(PA) have an alkaline pH, the SP(LS) has near to neutral pH, and only SP(PCE) solutions have an acidic pH, which retards Ca ions’ transition in the solution. Moreover, the retardation of the hydration process can be caused partly by the adsorption of SP molecules onto the surface of anhydrous cement compounds. The created layer of precipitated calcium binders on anhydrous alkaline cement compounds and the lower Ca^2+^ concentration in the system stop water from reaching the cement-particle surface and retard further hydration [83].

It can be concluded that each admixture used separately and especially admixture combinations when MWCNTs, AEA, and SPs are used together decrease cement dissolution and prolong initial setting time compared to the control paste. Admixtures with neutral or alkaline pH in the cement paste show faster setting times.

The final setting time in pastes without MWCNTs varies from 180–340 min, depending on the admixture used. In the pastes with MWCNTs only, with an increase in MWCNT amount in the paste, the final setting time increases from 180 to 400 min. The final setting time mostly prolongs with 0.05% and 0.5% MWCNT amounts, which means that such MWCNT amounts can significantly prolong the hydration process.

With the increase in MWCNT amount in pastes with AEA, the final setting time increases even more, from 200 to 390 min. In pastes with AEA and SP admixtures, an increase in MWCNT amount in the pastes prolongs the final setting time from 205 to 530 min. Significant prolongation of final setting time is observed when the MWCNT amount exceeds 0.005%. In comparison to the pastes with AEA only, the final setting time is prolonged by the following, in decreasing order: by SP(PCE) up to 2.7 times, then by SP(PA) up to 2.2 times, and finally by SP(LS) up to 2.0 times.

It is evident that the pH and EC of used AEA and SPs influence the rheological properties of the pastes, such as spread and setting time. It can be concluded that SP(PCE), characterized by acidic pH values—the lowest between tested SP admixture EC values— most prolongs the final setting time. Admixtures with neutral or alkali pH in the cement paste show faster setting times.

## 4. Conclusions

The foam stability in the presence of cement, MWCNTs, and different kinds of SPs was tested in this study. The role of the cement on the foamed suspension stability was explained.

Evaluating the stability of the foam allows us to conclude that catching air bubbles in the MWCNT-filament network increases the stability of foam, especially at the higher amounts of MWCNTs (0.00037–0.37 g). An increase in the amount of MWCNTs improves foam stability and increases the initial volume of foam. More significantly, between tested SPs (LS, PA, and PCE) in the foamed suspension with MWCNTs, the presence of SP(PA) increased the initial volume of foam, but the presence of SP(PCE) decreased the initial volume of foam.

It can be concluded that the addition of MWCNTs works to cause solid particle size effects, catches air in the filament structure and improves foam stability. However, the stability of foams greatly depends on the used SPs. SP(LS) contributes to foam stability more and SP(PCE) contributes less.

The viscosity test confirms the foam stability test results. With an increase in the amount of MWCNTs, the viscosity of pure water and foam increases due to synergetic interaction between the foam and the MWCNT filaments. When both AEA and MWCNT admixtures are used, their synergistic effect on viscosity is noticeable. When AEA and MWCNTs act together, the viscosity reaches 20 and 4.5 times higher compared to MWCNTs and AEA used separately, respectively. The presence of SP(LS) reduces the foam viscosity the least, and SP(PCE) reduces the viscosity the most.

The addition of cement increases the viscosity of the foam of the paste with MWCNTs by 2.6 times compared to the control cement-free sample. The addition of cement in the foam with MWCNTs of different types increased viscosity by up to 40% compared to the control cement-free sample.

The conductivity results of cement pastes with MWCNT additions show that an increase in MWCNT amount retarded the dissolution of cement minerals and decreased the paste’s EC. Adding AEA to cement paste with MWCNTs has minimal influence on the cement paste’s EC, whereas SP(LS) decreases the paste’s EC by 15%, the SP(PA) decreases it by 16.9%, and SP(PCE) decreases it by 24.2%. This indicates that, when combined with MWCNTs and AEA, these superplasticizers decrease the cement paste’s EC.

The conductivity results of cement pastes with different admixtures are supported by paste spread and setting time results. With increased amounts of MWCNTs in the foamed paste, spread values decrease to 15.25% compared to AEA-free pastes. The presence of SP(LS), SP(PA), and SP(PCE) improved the spread values of the paste to 3.8, 8.8, and 10.5%, respectively. With increased amounts of MWCNTs in the foamed paste, the initial setting time is prolonged up to 2.4 times.

The AEA admixture, characterized by the lowest electrical conductivity in water solution, prolongs the initial setting time of pastes with MWCNTs. The presence of SP(LS), SP(PA), and SP(PCE) prolong the initial setting time to 2.8 times with the highest MWCNT amount (0.5%) in comparison to the control paste with MWCNTs and AEA admixture.

## Figures and Tables

**Table 1:** The characteristics of SPs and AEA.

	Admixtures Type			
	SP(LS)	SP(PA)	SP(PCE)	AEA
pH	6.21	8.12	4.7	8.05
Electrical conductivity, S/m	2011 × 10^−4^	551 × 10^−4^	367 × 10^−4^	320 × 10^−4^
Density, g/cm^3^	1.14	1.06	1.06	1.30
Color	dark	yellow-brown	brown	white
Dry matter content, %	31.0	27.0	27.0	92
Molecular weight, g/mol	35.0	39.4	51.0	–

**Table 2:** Water suspension compositions with different SP types and different amounts of MWCNTs (g).

Batch	Cement	MWCNTs *	AEA *	SP(LS) *	SP(PA) *	SP(PCE) *	Water
W0	–	0	–	–	–	–	40
W1	–	0.000037	–	–	–	–	40
W2	–	0.00037	–	–	–	–	40
W3	–	0.0037	–	–	–	–	40
W4	–	0.037	–	–	–	–	40
W5	–	0.37	–	–	–	–	40
W0-A	–	0	0.03	–	–	–	40
W1-A	–	0.000037	0.03	–	–	–	40
W2-A	–	0.00037	0.03	–	–	–	40
W3-A	–	0.0037	0.03	–	–	–	40
W4-A	–	0.037	0.03	–	–	–	40
W5-A	–	0.37	0.03	–	–	–	40
W0-A-LS	–	0	0.03	0.2	–	–	40
W1-A-LS	–	0.000037	0.03	0.2	–	–	40
W2-A-LS	–	0.00037	0.03	0.2	–	–	40
W3-A-LS	–	0.0037	0.03	0.2	–	–	40
W4-A-LS	–	0.037	0.03	0.2	–	–	40
W5-A-LS	–	0.37	0.03	0.2	–	–	40
W0-A-PA	–	0	0.03	–	0.2	–	40
W1-A-PA	–	0.000037	0.03	–	0.2	–	40
W2-A-PA	–	0.00037	0.03	–	0.2	–	40
W3-A-PA	–	0.0037	0.03	–	0.2	–	40
W4-A-PA	–	0.037	0.03	–	0.2	–	40
W5-A-PA	–	0.37	0.03	–	0.2	–	40
W0-A-PCE	–	0	0.03	–	–	0.2	40
W1-A-PCE	–	0.000037	0.03	–	–	0.2	40
W2-A-PCE	–	0.00037	0.03	–	–	0.2	40
W3-A-PCE	–	0.0037	0.03	–	–	0.2	40
W4-A-PCE	–	0.037	0.03	–	–	0.2	40
W5-A-PCE	–	0.37	0.03	–	–	0.2	40
C-W0-A	8	0	0.03	–	–	–	40
C-W1-A	8	0.000037	0.03	–	–	–	40
C-W2-A	8	0.00037	0.03	–	–	–	40
C-W3-A	8	0.0037	0.03	–	–	–	40
C-W4-A	8	0.037	0.03	–	–	–	40
C-W5-A	8	0.37	0.03	–	–	–	40
C-W0-A-LS	8	0	0.03	0.2	–	–	40
C-W1-A-LS	8	0.000037	0.03	0.2	–	–	40
C-W2-A-LS	8	0.00037	0.03	0.2	–	–	40
C-W3-A-LS	8	0.0037	0.03	0.2	–	–	40
C-W4-A-LS	8	0.037	0.03	0.2	–	–	40
C-W5-A-LS	8	0.37	0.03	0.2	–	–	40
C-W0-A-PA	8	0	0.03	–	0.2	–	40
C-W1-A-PA	8	0.000037	0.03	–	0.2	–	40
C-W2-A-PA	8	0.00037	0.03	–	0.2	–	40
C-W3-A-PA	8	0.0037	0.03	–	0.2	–	40
C-W4-A-PA	8	0.037	0.03	–	0.2	–	40
C-W5-A-PA	8	0.37	0.03	–	0.2	–	40
C-W0-A-PCE	8	0	0.03	–	–	0.2	40
C-W1-A-PCE	8	0.000037	0.03	–	–	0.2	40
C-W2-A-PCE	8	0.00037	0.03	–	–	0.2	40
C-W3-A-PCE	8	0.0037	0.03	–	–	0.2	40
C-W4-A-PCE	8	0.037	0.03	–	–	0.2	40
C-W5-A-PCE	8	0.37	0.03	–	–	0.2	40

* above the dry matter content.

**Table 3:** Cement-paste compositions with different SP types and different amounts of MWCNTs (%).

Batch	Cement	MWCNTs *	AEA *	SP(LS) *	SP(PA) *	SP(PCE) *	W/C Ratio
K-C0	100	0	–	–	–	–	0.27
K-C1	100	0.00005	–	–	–	–	0.27
K-C2	100	0.0005	–	–	–	–	0.27
K-C3	100	0.005	–	–	–	–	0.27
K-C4	100	0.05	–	–	–	–	0.27
K-C5	100	0.5	–	–	–	–	0.27
K-C0-A	100	0	0.03	–	–	–	0.27
K-C1-A	100	0.00005	0.03	–	–	–	0.27
K-C2-A	100	0.0005	0.03	–	–	–	0.27
K-C3-A	100	0.005	0.03	–	–	–	0.27
K-C4-A	100	0.05	0.03	–	–	–	0.27
K-C5-A	100	0.5	0.03	–	–	–	0.27
K-C0-A-LS	100	0	0.03	0.2	–	–	0.27
K-C1-A-LS	100	0.00005	0.03	0.2	–	–	0.27
K-C2-A-LS	100	0.0005	0.03	0.2	–	–	0.27
K-C3-A-LS	100	0.005	0.03	0.2	–	–	0.27
K-C4-A-LS	100	0.05	0.03	0.2	–	–	0.27
K-C5-A-LS	100	0.5	0.03	0.2	–	–	0.27
K-C0-A-PA	100	0	0.03	–	0.2	–	0.27
K-C1-A-PA	100	0.00005	0.03	–	0.2	–	0.27
K-C2-A-PA	100	0.0005	0.03	–	0.2	–	0.27
K-C3-A-PA	100	0.005	0.03	–	0.2	–	0.27
K-C4-A-PA	100	0.05	0.03	–	0.2	–	0.27
K-C5-A-PA	100	0.5	0.03	–	0.2	–	0.27
K-C0-A-PCE	100	0	0.03	–	–	0.2	0.27
K-C1-A-PCE	100	0.00005	0.03	–	–	0.2	0.27
K-C2-A-PCE	100	0.0005	0.03	–	–	0.2	0.27
K-C3-A-PCE	100	0.005	0.03	–	–	0.2	0.27
K-C4-A-PCE	100	0.05	0.03	–	–	0.2	0.27
K-C5-A-PCE	100	0.5	0.03	–	–	0.2	0.27

* above the dry matter content.

## Data Availability

Data are contained within the article.
